# Predicting Sweetness Intensity and Uncovering Quantitative Interactions of Mixed Sweeteners: A Machine Learning Approach

**DOI:** 10.3390/foods15010167

**Published:** 2026-01-04

**Authors:** Tiantian Du, Gang He, Xin Hou, Peiqin Shi, Zhilei Zhou, Jian Mao

**Affiliations:** 1 National Engineering Research Center of Cereal Fermentation and Food Biomanufacturing, School of Food Science and Technology, Jiangnan University, Wuxi 214122, China; 6230111024@stu.jiangnan.edu.cn (T.D.); ggang830@163.com (G.H.); houxinu@126.com (X.H.); 7240112088@stu.jiangnan.edu.cn (P.S.); 2 Jiangnan University (Shaoxing) Industrial Technology Research Institute, Shaoxing 312000, China; 3 National Engineering Research Center for Huangjiu, Shaoxing 312000, China

**Keywords:** sweetener, synergy, interaction, sweetness intensity, machine learning

## Abstract

Sweeteners are commonly blended to exploit synergistic effects, enabling the desired sweetness to be attained while reducing total usage. However, establishing a quantitative relationship between mixed sweeteners’ concentration and sweetness intensity remains a key challenge. This study developed a sensory evaluation–machine learning approach to construct prediction models for binary/ternary mixtures of five sweeteners (sucrose, glucose, fructose, mannitol, and sorbitol). After feature selection of molecular descriptors and comparison of seven machine learning regression models, the Multilayer Perceptron achieved superior performance for the binary mixtures (R^2^ = 0.9828), while the Support Vector Regression exhibited optimal performance for the ternary mixtures (R^2^ = 0.9825). Concentration–sweetness intensity curves of mixed sweeteners at specific concentrations were generated using these two optimal prediction models. Results showed that at low concentrations, ternary blends of one sugar and two polyols (mannitol and sorbitol) exhibited stronger synergism than binary mixtures in the same concentration range. Specifically, blending the composite system of 1% mannitol and 2% sorbitol with 1% sucrose, 1% glucose, and 1% fructose separately increased the sweetness intensity by 39.6%, 42.8%, and 37.4%, respectively. This work confirms that machine learning can establish a quantitative relationship between multi-component sweeteners’ concentration and sweetness intensity, reveal their complex interactions, and provide a novel approach for intelligent sensory assessment and formulation design.

## 1. Introduction

Sweeteners represent a foundational class of food additives [1,2], characterized by diverse types and extensive applications across a wide range of food products [3]. However, reliance on any single sweetener is often constrained by specific drawbacks. For instance, the overconsumption of widely used sucrose is linked to adverse health outcomes, including obesity and diabetes [4]. Conversely, many high-intensity sweeteners (e.g., stevioside) can impart undesirable sensory attributes, such as lingering aftertastes or bitterness [5], thereby compromising the overall flavor profile. To address these challenges, the blending of sweeteners has become a common practice in modern food and beverage formulation [6,7,8,9]. Blending different sweeteners may produce a synergistic effect, which not only reduces the dosage of individual sweeteners and lowers production costs but also significantly improves the sweetness quality of food products [10]. Moreover, the combined application of sweeteners can mitigate the sensory deficiencies of single sweeteners, thereby providing consumers with a superior sensory experience [11,12].

The coexistence of multiple sweeteners elicits complex interactions (i.e., synergistic, additive, and antagonistic effects), which in turn modulate human perceptual responses to food sweetness. To date, numerous binary sweetener combinations with synergistic effects have been reported. Notably, high-intensity sweeteners tend to exhibit strong synergistic effects when blended, such as the binary mixtures of acesulfame-K (Ace-K) and aspartame; neohesperidin dihydrochalcone (NHDC) and sucralose; rebaudioside A (Reb A) and Ace-K; as well as Reb A and sucralose [13,14]. Furthermore, synergistic effects are not limited to high-intensity sweetener combinations, but also occur between saccharides, polyols, and high-intensity sweeteners, as exemplified by combinations of sorbitol with sweeteners such as sucrose, fructose, and Ace-K [15]. Compared to binary systems, the interactions in multi-component blends are considerably more complex and remain significantly underexplored [16]. Building on synergistic binary sweetener combinations from previous studies, researchers have conducted ternary blending experiments and demonstrated that the mixture of alitame, NHDC, and Reb A exhibited the strongest synergism among all tested combinations [17]. Currently, with respect to the perceptual quantification of sweetness intensity for mixed sweeteners, only a handful of studies have clarified the dose–response relationship between individual component concentrations and resultant sweetness intensity. Research on the quantitative relationship between a broader range of sweetener concentrations and sweetness intensity remains scarce, leaving the dose-dependent association between mixed sweetener concentrations and sweetness intensity yet to be fully elucidated.

With the rapid advancement of machine learning (ML) technology, relevant intelligent analytical approaches have been successfully applied to the field of food flavor prediction in recent years [18,19]. A key advantage of ML lies in its ability to efficiently analyze large, complex datasets and accurately model intricate nonlinear relationships, which enables objective, consistent, and rapid evaluation of food sensory quality to achieve precise prediction [20]. Currently, leveraging ML models to predict the sweetness of compounds [21,22,23,24] and integrating them with multi-electronic sensory technology [25], metabolomics [26], and flavoromics [27] to forecast consumer preferences and sensory attributes has emerged as a crucial technical strategy in the field of flavor prediction.

In this study, sensory evaluation experiments were conducted to formulate binary and ternary blends of five sweeteners and quantify their corresponding sweetness intensities. Seven ML models were integrated to develop predictive models for the sweetness intensity of these binary and ternary sweetener mixtures. These regression models were subsequently utilized to predict the sweetness intensity of mixed sweeteners across a specified concentration range. Furthermore, the Shapley Additive exPlanations (SHAP) analysis method was employed to identify the key variables governing the sweetness intensity of mixed sweeteners. This work elucidates the synergistic or antagonistic patterns arising from sweetener combinations and presents a novel approach for the quantitative analysis of sweetness perception in multi-sweetener systems.

## 2. Materials and Methods

### 2.1. Materials and Chemical

Sucrose (≥98%), glucose (≥99%), fructose (≥99%), mannitol (≥99%), and sorbitol (≥99%) were food-grade reagents provided by Laibin Dongtang Fenghuang Co., Ltd. (Laibin, China), Xiwang Pharmaceutical Co., Ltd. (Zouping, China), Xiwang Sugar Industry Co., Ltd. (Zouping, China), Mingyue Seaweed Group Co., Ltd. (Qingdao, China), and Huakang Pharmaceutical Co., Ltd. (Quzhou, China), respectively.

### 2.2. Establishment of the Sensory Evaluation Panel

Sensory panelists were recruited from graduate students of the School of Food Science and Technology, Jiangnan University, all of whom volunteered to participate and possessed prior experience in sensory evaluation. Panelist qualification prescreening was conducted in accordance with ISO 8586:2023 [28], in compliance with sensory quality control criteria, to initially identify candidates with high sensitivity to sweetness and no distinct preferences or aversions toward the test samples. In line with ISO 4120:2021 [29] and ISO 8587:2006 [30], the shortlisted candidates underwent rigorous secondary screening and systematic training. Throughout the training period, panelists’ performance was monitored and evaluated using Panel Check 1.4.2 software to assess their discriminatory ability, repeatability, and consistency. Ultimately, 12 panelists (aged 25 ± 3 years, 6 males and 6 females) who demonstrated satisfactory performance across all three metrics were selected to form the official sensory evaluation panel.

Formal training was further administered to the selected panel members. Sucrose solutions with varying concentrations were prepared, and panelists were instructed to score these samples using a predefined rating scale, with the aim of ensuring they proficiently mastered the sensory evaluation workflow and standardized operation of the sweetness scoring criteria. The training program was conducted three times weekly, 1 h per session, for a total duration of two months. Upon completion of training, a preliminary experiment was conducted, where each sample was evaluated by the panelists for at least three replications. The formal experiment was initiated only when the panelists achieved stable performance in repeatability, consistency, and discriminatory ability for sweetness intensity scoring (detailed performance metrics are presented in Figure S1, including F plot, MSE plot, and Profile plot).

### 2.3. Sample Preparation

Individual stock solutions of the five target sweeteners were prepared at the concentrations specified in Table S1. Binary and ternary sweetener blends were formulated in accordance with the concentrations listed in Table 1, with the total sweetener concentration of each blend capped at 15.0% (excluding fructose-containing blends). Specifically, 10 binary blend combinations were designed, with 22 concentration gradients set for each combination, yielding a total of 220 individual samples. For ternary blends, 10 distinct combinations were prepared, each with 46 concentration gradients, resulting in a total of 460 samples.

Sucrose, a widely utilized reference standard for sweetness evaluation, was selected as the reference substance in accordance with ISO 3972:2011 [31]. A 0–15 point scoring scale was adopted for sweetness quantification: sucrose solutions with weight/volume (*w*/*v*) concentrations of 2.0%, 5.0%, 7.5%, 10.0%, 12.0%, and 16.0% were assigned sweetness scores of 2.0, 5.0, 7.5, 10.0, 12.0, and 15.0, respectively, to establish a series of reference standards with gradient sweetness intensities. All samples were prepared using purified water (Wahaha Group Co., Ltd., Hangzhou, China) at room temperature (22 ± 2 °C) for 5 h prior to sensory evaluation.

### 2.4. Sensory Evaluation Procedure

Trained sensory panelists were instructed to score the sweetness intensity of individual sweeteners and binary/ternary sweetener blends using a 0–15 point scoring scale, with five samples assessed per test session. All samples were presented in transparent, odorless tasting cups and labeled with random three-digit codes. Panelists were required to consume 15 mL of each sample per evaluation, and each sample was assessed in triplicate to ensure result reliability. Before tasting the next sample, panelists were instructed to rinse their mouths with purified water and consume salt-reduced soda biscuits to eliminate residual tastes; a 5 min rest interval was set between consecutive samples to mitigate sensory fatigue. The final sensory score for each sample was calculated as the mean value of triplicate scores from all panelists. To avoid interference with sensory perception, panelists were required to abstain from consuming any food or beverages other than water for 30 min prior to each test session. All sensory evaluations were conducted in the standard sensory booths at Jiangnan University, which comply with the test room design specifications outlined in ISO 8589:2007 [32].

### 2.5. Methods to Determine the Synergistic Effect of Sweetener Mixtures

The sweetness intensity of binary and ternary sweetener blends was compared with the theoretical total sweetness intensity of their individual unmixed components. Specifically, a sweetener mixture was classified as exhibiting a synergistic, additive, or antagonistic effect if its measured sweetness intensity was significantly higher than, approximately equivalent to, or notably lower than the total sweetness intensity of its individual components, respectively. This approach is the most common and straightforward approach for evaluating sweetener interaction effects, and it has been widely adopted in relevant studies on sweetener compounding [9,17].

### 2.6. Dataset Processing

Simplified molecular input line entry system (SMILES) strings of the five sweeteners’ chemical structures were retrieved from the PubChem database (https://pubchem.ncbi.nlm.nih.gov/, accessed on 7 May 2025). Subsequently, three types of core information (SMILES strings of the sweeteners, concentration values of the five sweeteners in each blend (Table 2), and the corresponding sweetness intensity results of the mixtures) were compiled into an Excel spreadsheet. This dataset served as the foundational input for the subsequent development of machine learning models. The meanings of partial molecular descriptors in the dataset are presented in Table 2.

### 2.7. Calculation of Molecular Descriptors and Feature Processing

Molecular descriptors of the five sweeteners were calculated using the open-source chemoinformatics software RDKit (https://www.rdkit.org/, accessed on 14 May 2025), version 2024.03.1, with the detailed calculation process shown in Figure S2, and the molecular descriptor results for each sweetener are presented in Table S2. The concentration of each sweetener component was incorporated into the model as an independent feature, which was combined with the molecular descriptors to form the final input vector. Prior to model training, all features were subjected to Z-score standardization using StandardScaler, with the calculation formula shown in Equation (1):

Z = (X − μ)/σ(1)

where X represents the original value of the feature, μ denotes the mean value of the feature in the training set, and σ is the standard deviation of the feature in the training set. This standardization process ensures all features have a mean of 0 and a standard deviation of 1, which eliminates dimensional discrepancies between different descriptors and improves both the convergence speed and stability of the machine learning model.

### 2.8. Regression Modeling and Model Evaluation

Seven regression algorithms were employed to predict the sweetness intensity of binary and ternary sweetener blends: Adaptive Boosting (AdaBoost), Light Gradient Boosting Machine (LightGBM), Random Forest (RF), Gradient Boosting Decision Tree (GBDT), Multilayer Perceptron (MLP), eXtreme Gradient Boosting (XGBoost), and Support Vector Regression (SVR). Among these, AdaBoost [33], LightGBM [34], GBDT [35], and XGBoost [36] all belong to the category of boosting ensemble algorithms, whose core principle is to construct a strong learner by iteratively optimizing and combining multiple weak learners. RF leverages its advantages of overfitting resistance and simple parameter tuning to effectively establish complex nonlinear relationships by integrating multiple decision trees [37,38]. In contrast, SVR inherits the core idea of Support Vector Machines (SVMs) and exhibits excellent generalization ability in numerous regression tasks, with strong resistance to overfitting [39]. MLP is a feedforward artificial neural network model that learns complex mapping relationships in data by adjusting the weights between neurons, thereby solving prediction problems [40].

All samples were randomly split into training and test sets at an 8:2 ratio. The training set was used to train the seven aforementioned models, with hyperparameters optimized via grid search combined with 5-fold cross-validation. Finally, these models were used to predict the test set, and the performance of the seven models was evaluated based on four evaluation metrics: determination coefficient (R^2^), root mean square error (RMSE), mean square error (MSE), and mean absolute error (MAE). A higher R^2^ value and lower RMSE, MSE, and MAE values indicate better fitting and predictive performance of the model. The hyperparameters used for each model during grid search are detailed in Table S3.

### 2.9. Explanatory Analysis of Feature Importance

Feature importance analysis and the SHAP method were employed to systematically evaluate the impact of input variables on the final prediction results. Feature importance analysis assesses the influence of each variable on prediction outcomes by quantifying its weight coefficient. In contrast, the SHAP method, which is grounded in Shapley values from game theory, aims to precisely calculate the specific contribution of each variable to model predictions, thereby providing more detailed interpretations [41]. These two methods were further utilized to screen the molecular descriptors input into the models, so as to further optimize the model performance. Specifically, the absolute value of the SHAP contribution was defined as the sole quantitative criterion for screening: a larger absolute value indicates a higher contribution of the descriptor to the model prediction, while an absolute value close to 0 denotes a redundant feature that was excluded. This screening strategy achieves feature dimensionality reduction while ensuring the predictive accuracy of the models.

### 2.10. Statistical Analysis of Data

The selection and performance of panelists were evaluated using Panel Check 1.4.2 software. All machine learning models were established using Python 3.8 on the Jupyter Notebook 7.2.2 platform, and all data processing was implemented on the Anaconda3 platform. Specific Python libraries were utilized for targeted tasks: pandas 2.2.2 was used for data reading and preprocessing; NumPy 1.26.4 for numerical calculations and array operations; scikit-learn 1.5.1 for dataset splitting, feature standardization, model training, hyperparameter tuning, and model performance evaluation; matplotlib 3.9.2 for the visualization of experimental results; and shap 0.46.0 for the implementation of SHAP-based feature importance analysis.

## 3. Results and Discussion

### 3.1. Establishment of Prediction Models for Sweetness Intensity of Binary Sweeteners

#### 3.1.1. Regression Model Performance Evaluation for Binary Sweeteners

In terms of the R^2^ metric, the GBDT, MLP, XGBoost, and SVR models exhibited superior performance; their R^2^ values for both the training and test sets exceeded 0.95 (Table 3), demonstrating a high degree of consistency between predicted and measured sweetness intensity values.

**Figure 1:**
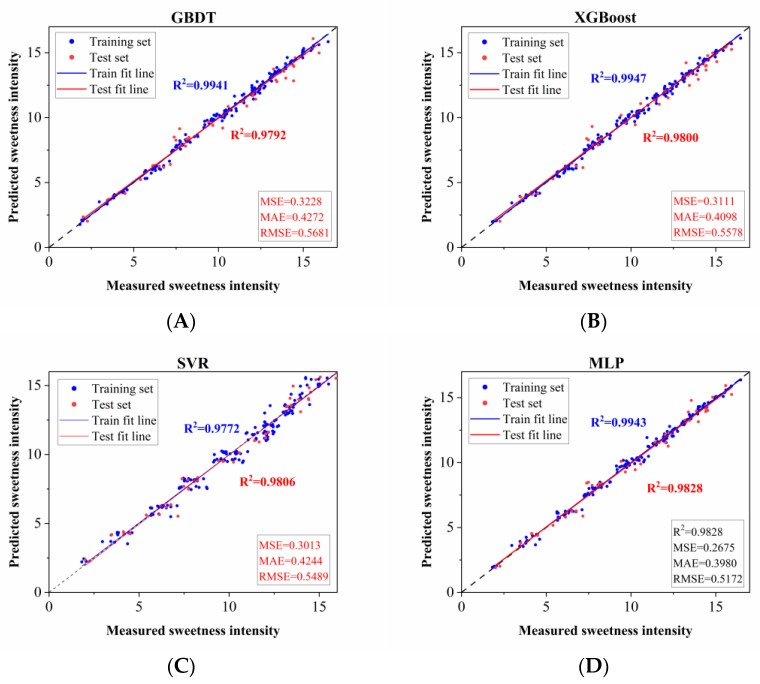
Prediction models for sweetness intensity of binary sweeteners based on (**A**) GBDT, (**B**) XGBoost, (**C**) SVR, and (**D**) MLP algorithms after molecular descriptor screening. X-axis: measured sweetness intensity (from sensory evaluation by the trained panel); Y-axis: predicted sweetness intensity by the models.

To further improve model efficiency and interpretability, feature importance analysis was conducted to quantify the influence of each molecular descriptor on the models’ predictive outcomes. Based on the results of the SHAP method for all molecular descriptors (details in Table S4), we filtered the input molecular descriptors by reducing the descriptor dimensionality while maintaining the predictive accuracy of the models, and further optimized the four high-performance models (GBDT, MLP, XGBoost, and SVR). Specifically, the optimized MLP, GBDT, XGBoost, and SVR models adopted 195, 25, 20, and 90 molecular descriptors, respectively. The optimal performance results of these four models are visualized in Figure 1. Among the four machine learning models (GBDT, XGBoost, SVR, and MLP), the MLP model exhibited the best comprehensive prediction performance, achieving the highest test set R^2^ value (0.9828) and the lowest MAE (0.3980) and RMSE values among all models. Additionally, the MSE and RMSE of the MLP model were lower than those of the other three models (MSE: 0.2675, RMSE: 0.5172), confirming that the MLP model exhibited the best performance. This result may be attributed to the core characteristics of MLP, which models and learns complex data relationships through its multilayer neural network structure and nonlinear activation functions [42]. Therefore, the MLP model was selected as the optimal model for predicting the sweetness intensity of binary sweeteners in subsequent experiments.

#### 3.1.2. Interpretable Machine Learning: SHAP Feature Analysis for Binary Sweeteners

**Figure 2:**
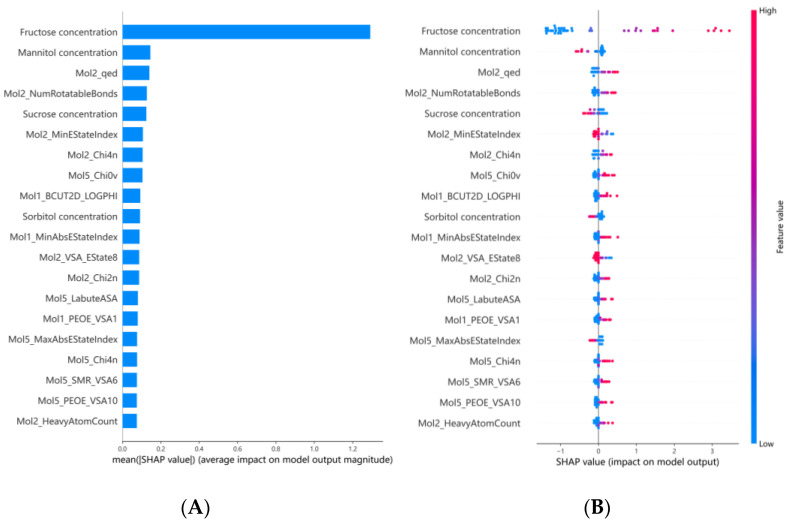
SHAP analysis of descriptors’ influence on the MLP model: (**A**) feature importance ranking, (**B**) SHAP summary plot. Horizontal axis: SHAP values (indicating the magnitude and direction of feature impact; positive = promoting, negative = inhibiting sweetness intensity). Vertical axis: molecular descriptors ranked by predictive weight. Point color: pink = high feature value, blue = low feature value. Mol1: sucrose; Mol2: glucose; Mol5: sorbitol.

To clarify the relationship between relevant molecular descriptors and the sweetness intensity of binary sweeteners, the SHAP method was applied to interpret the optimal MLP model. The SHAP values of individual features and their contribution rates to the model’s predictions were calculated; the results for the top 20 features are visualized in Figure 2, and the specific contribution rates of all features are presented in Table S5. In Figure 2A, the feature importance of the MLP model is ranked using mean absolute Shapley values. The results indicated that fructose concentration was identified as the most critical descriptor, followed by mannitol concentration and Mol2_qed (quantitative estimate of drug-likeness). Figure 2B presents a summary plot that illustrates feature importance and feature effects on prediction outcomes. Specifically, high Shapley values of fructose concentration exerted a significant positive impact on the model’s predicted sweetness intensity. For mannitol concentration, low Shapley values exhibited positive effects on the predictions, while high values exerted negative impacts. In contrast, high Shapley values of Mol2_qed showed positive influences on the model’s output. These results indicated that fructose concentration exerted the most significant impact on the sweetness intensity of binary sweeteners, while mannitol concentration had a relatively minor effect. This may be attributed to the fact that among the five sweeteners, fructose exhibited the highest sweetness intensity at the same weight/volume concentration, whereas mannitol had a relatively low sweetness intensity. Notably, systematic evaluation of binary mixtures of fourteen sweeteners confirmed that fructose produced significant sweetness synergism when compounded with high-intensity sweeteners such as acesulfame-K and Na saccharin, with the degree of synergism varying with the concentration ratio of the blended components [15]. Additionally, the molecular descriptor Mol2_qed is a comprehensive quantitative score of drug-likeness derived from integrating multiple drug-related properties such as molecular weight, LogP, and the number of hydrogen bond donors/acceptors [43], suggesting a potential correlation between the quantitative estimate of drug-likeness (Qed) and sweetness perception.

### 3.2. Analysis of Concentration–Sweetness Intensity Curves for Binary Sweeteners

**Figure 3:**
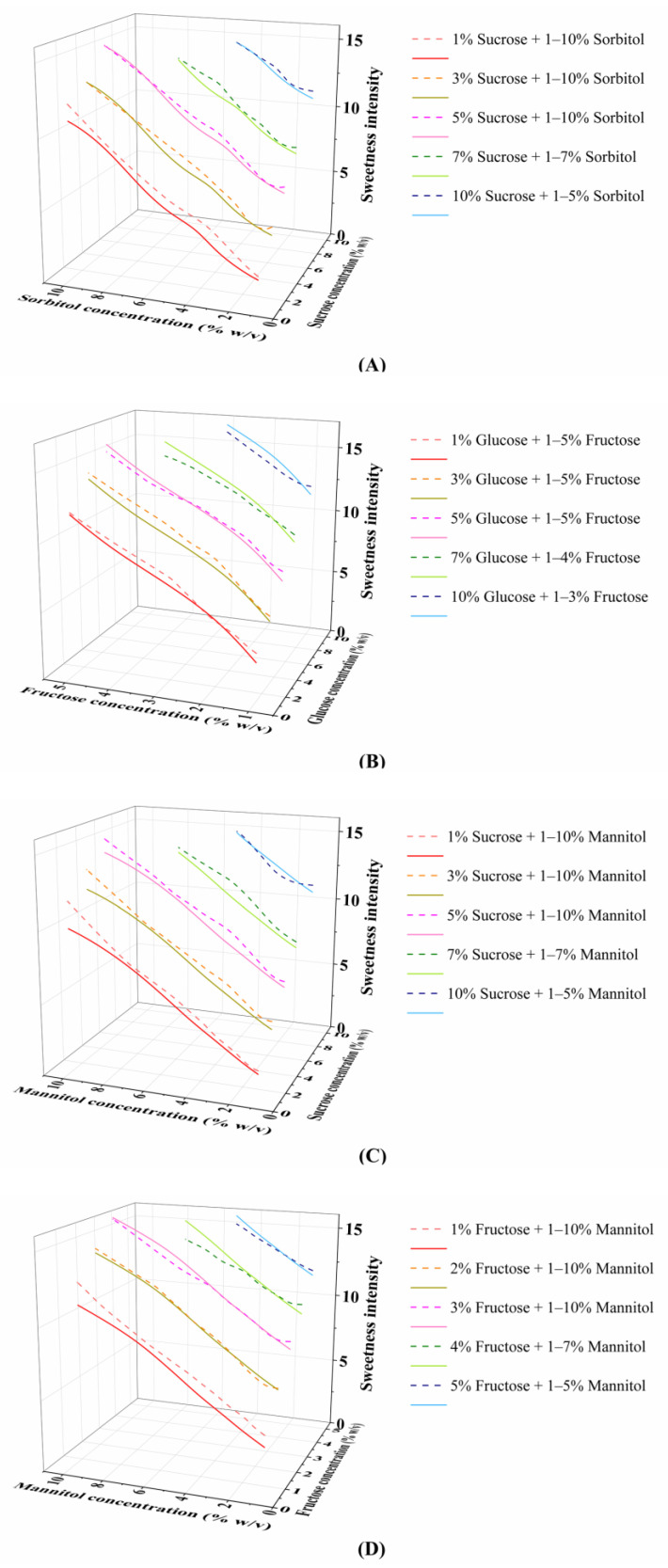
Concentration–sweetness intensity curves of binary sweeteners within a specific concentration range: (**A**) sucrose and sorbitol; (**B**) glucose and fructose; (**C**) sucrose and mannitol; (**D**) fructose and mannitol. The dashed lines represent the sweetness intensity of binary mixtures, and the solid lines represent the direct sum of the sweetness intensities of the individual components. A synergistic effect is indicated when the dashed line of the same color lies above the solid line.

The sweetness intensity resulting from binary sweetener concentrations within the range typically used in beverages [44] was predicted using the optimized MLP model. To elucidate the quantitative concentration–sweetness interaction relationships, representative concentration combinations were selected to plot the corresponding relationship curves (Figure 3).

Concentration was identified as a key factor governing synergistic and antagonistic effects in binary sweetener mixtures. Synergism predominated in low-concentration mixtures and gradually diminished with increasing component concentrations, in agreement with previous findings [15,45]. A typical example is the sucrose–sorbitol mixture (Figure 3A): at 1.0% concentration for both components, the mixture exhibited a 13.7% sweetness enhancement relative to the theoretical additive value; the synergism weakened to 6.6% at 5.0% and further declined to 0.9% at 7.0%, indicating a shift to an additive effect. This trend was validated by the glucose–sorbitol mixture (Figure S3A), where the sweetness increment decreased from 17.7% at 3.0% to 1.4% at 7.0%. Notably, some binary mixtures underwent a complete transition from synergism to antagonism at high concentrations. As shown in Figure S3B, the fructose–sorbitol mixture achieved a 29.5% sweetness enhancement at 1.0% fructose combined with 2.0% sorbitol, but shifted to 4.1% antagonism at 3.0% fructose and 7.0% sorbitol.

Sweetener type also exerted a marked influence on interaction patterns, with two distinct response trends observed in binary sweetener mixtures. Notably, sucrose is inherently a disaccharide composed of one molecule of glucose and one molecule of fructose. This inherent property underpins the weak synergism observed in sugar–sugar binary mixtures. Binary mixtures composed of sucrose, glucose, and fructose exhibited weaker synergism and a consistent transition from synergism to antagonism with increasing concentrations. For the glucose–fructose mixture (Figure 3B), fixing glucose at 5.0% resulted in 10.5% and 3.1% synergistic increments with 1.0% and 2.0% fructose, respectively; however, antagonism occurred at 4.0% and 5.0% fructose, with sweetness intensity decreasing by 3.1% and 3.5%, respectively, and the interaction transition concentration was 3.0% fructose. Similar concentration-dependent transitions were observed in sucrose-glucose mixtures (Figure S3C) and sucrose–fructose mixtures (Figure S3D), with antagonism occurring when glucose or fructose concentrations exceeded 5.0% and 2.0%, respectively, at a fixed sucrose concentration. In contrast, sugar–polyol mixtures (e.g., fructose–mannitol, glucose–sorbitol, sucrose–sorbitol) displayed significantly stronger synergism in the low-concentration range (1.0–3.0%): fructose–mannitol mixtures achieved 20.7–30.7% sweetness increments at 1.0% fructose with 1.0–3.0% mannitol (Figure 3D); glucose–sorbitol mixtures reached a maximum 33.3% increment (Figure S3A); and sucrose–sorbitol mixtures showed 28.3–29.4% enhancement (Figure 3A). These results confirm that low-concentration sugar–polyol mixtures are not only more prone to synergism but also exhibit a significantly higher degree of synergism than sugar–sugar mixtures.

The divergent interaction patterns between sugar–sugar and sugar–polyol mixtures may be attributed to their distinct modes of engagement with the sweet taste receptor. For sugar–sugar mixtures, antagonism observed at higher concentrations could arise from steric hindrance or negative allosteric modulation within or near the primary sugar-binding pocket in the Venus flytrap (VFT) domain of T1R2. This might limit the optimal binding or signal transduction of individual components, thereby reducing overall receptor activation efficiency. In contrast, the robust synergy in sugar–polyol mixtures suggests a complementary binding mechanism. As supported by studies on polyol binding [46], polyols like mannitol and sorbitol likely engage distinct allosteric sites on the T1R2/T1R3 heterodimer. This allows sugars and polyols to bind concurrently and non-competitively, potentially stabilizing the active receptor conformation through cooperative interactions across subunits, thereby amplifying the sweet signal [14]. In summary, these findings demonstrated that both concentration and sweetener type are key factors regulating the interaction patterns of binary sweetener mixtures, with low-concentration sugar–polyol mixtures exhibiting the strongest synergistic potential.

### 3.3. Establishment of Prediction Models for Sweetness Intensity of Ternary Sweeteners

#### 3.3.1. Regression Model Performance Evaluation for Ternary Sweeteners

Consistent with the establishment of the binary sweetener sweetness intensity prediction model, AdaBoost, LightGBM, RF, MLP, GBDT, XGBoost, and SVR were adopted to construct prediction models for the sweetness intensity of ternary sweeteners. A 5-fold cross-validation strategy was implemented to assess model stability during the construction process. Based on the R^2^ metric, MLP, GBDT, XGBoost, and SVR performed best (Table 4), with R^2^ values of both the training and test sets exceeding 0.90, demonstrating excellent predictive performance.

**Figure 4:**
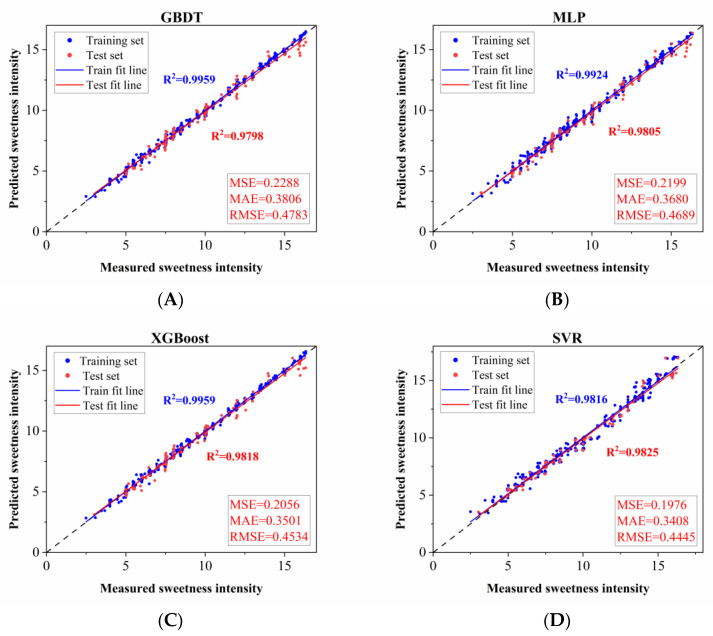
Prediction models for sweetness intensity of ternary sweeteners based on (**A**) GBDT, (**B**) MLP, (**C**) XGBoost, and (**D**) SVR algorithms after molecular descriptor screening. X-axis: measured sweetness intensity (from sensory evaluation by the trained panel); Y-axis: predicted sweetness intensity by the models.

To achieve further improvements in model efficiency and interpretability, the SHAP method was employed to quantify the influence of each input feature on model predictions (details in Table S6). Using these results, the input molecular descriptors were screened to further optimize the four high-performance models (MLP, GBDT, XGBoost, and SVR). Specifically, the optimized MLP, GBDT, XGBoost, and SVR models adopted 135, 70, 10, and 30 molecular descriptors, respectively. The performance analysis results of the four optimized models are presented in Figure 4. Among these models, the SVR model exhibited the most outstanding comprehensive performance when evaluating regression results based on molecular descriptors: it achieved the highest test set R^2^ value (0.9825) and the lowest MSE (0.1976), MAE (0.3408), and RMSE (0.4445), confirming its minimal prediction error. This advantage may stem from the kernel function mechanism of the SVR model, which can efficiently capture the nonlinear relationship between the concentrations of ternary sweeteners and sweetness intensity [47]. Overall, the SVR model demonstrated excellent predictive capability for the sweetness intensity of ternary sweeteners. Therefore, the SVR model was ultimately selected for predicting the sweetness intensity of ternary sweeteners.

#### 3.3.2. Interpretable Machine Learning: SHAP Feature Analysis for Ternary Sweeteners

**Figure 5:**
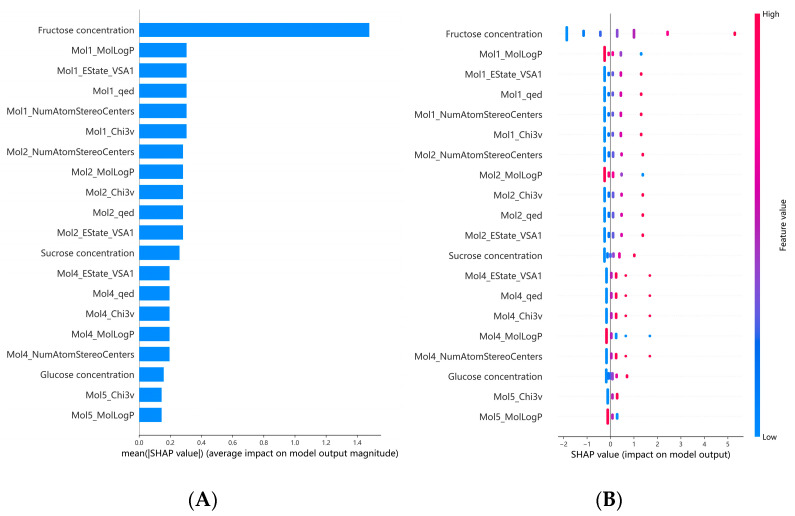
SHAP analysis of descriptors’ influence on the SVR model: (**A**) feature importance ranking, (**B**) SHAP summary plot. Horizontal axis: SHAP values (indicating the magnitude and direction of feature impact; positive = promoting, negative = inhibiting sweetness intensity). Vertical axis: molecular descriptors ranked by predictive weight. Point color: pink = high feature value, blue = low feature value. Mol1: sucrose; Mol2: glucose; Mol4: mannitol; Mol5: sorbitol.

To identify the molecular descriptors correlated with the sweetness intensity of ternary sweeteners, SHAP feature analysis was performed on the optimized SVR model. The SHAP values of individual features and their contribution rates to the model were calculated; the results for the top 20 features are visualized in Figure 5, and the specific contribution rates of all features are presented in Table S7. In Figure 5A, the feature importance of the SVR model is ranked using mean absolute Shapley values. The results indicated that fructose concentration was the most influential feature. Additionally, Mol1_MolLogP and Mol1_Estate_VSA1 also exhibited a certain degree of contribution to the model output. In Figure 5B, the summary plot illustrates the feature importance and its effects on prediction outcomes. Specifically, high Shapley values of fructose concentration exerted a significant positive impact on the model. For Mol1_MolLogP, lower feature values exerted a positive effect, whereas higher values exerted a negative effect. Meanwhile, high Shapley values of Mol1_Estate_VSA1 also exerted a positive impact on the model. In summary, fructose concentration had the most significant influence on the sweetness intensity of ternary sweeteners. This might be attributed to the fact that fructose has the highest sweetness intensity at equivalent weight/volume concentrations, which is consistent with the results of binary sweetener combinations. It is evident that in ternary combinations, fructose concentration also contributed substantially to the overall sweetness intensity. Additionally, MolLogP is a parameter that quantifies the distribution capacity of molecules between n-octanol and water, serving as an indicator of molecular hydrophobicity. Estate_VSA1 focuses on reflecting the charge distribution and hydrophilic–hydrophobic properties of local molecular regions, which may affect interactions with sweet taste receptors (e.g., hydrogen bond formation, electrostatic interactions). This indicates that hydrophobic interactions and electrostatic interactions are closely associated with sweetness perception [48].

### 3.4. Analysis of Concentration–Sweetness Intensity Curves for Ternary Sweeteners

**Figure 6:**
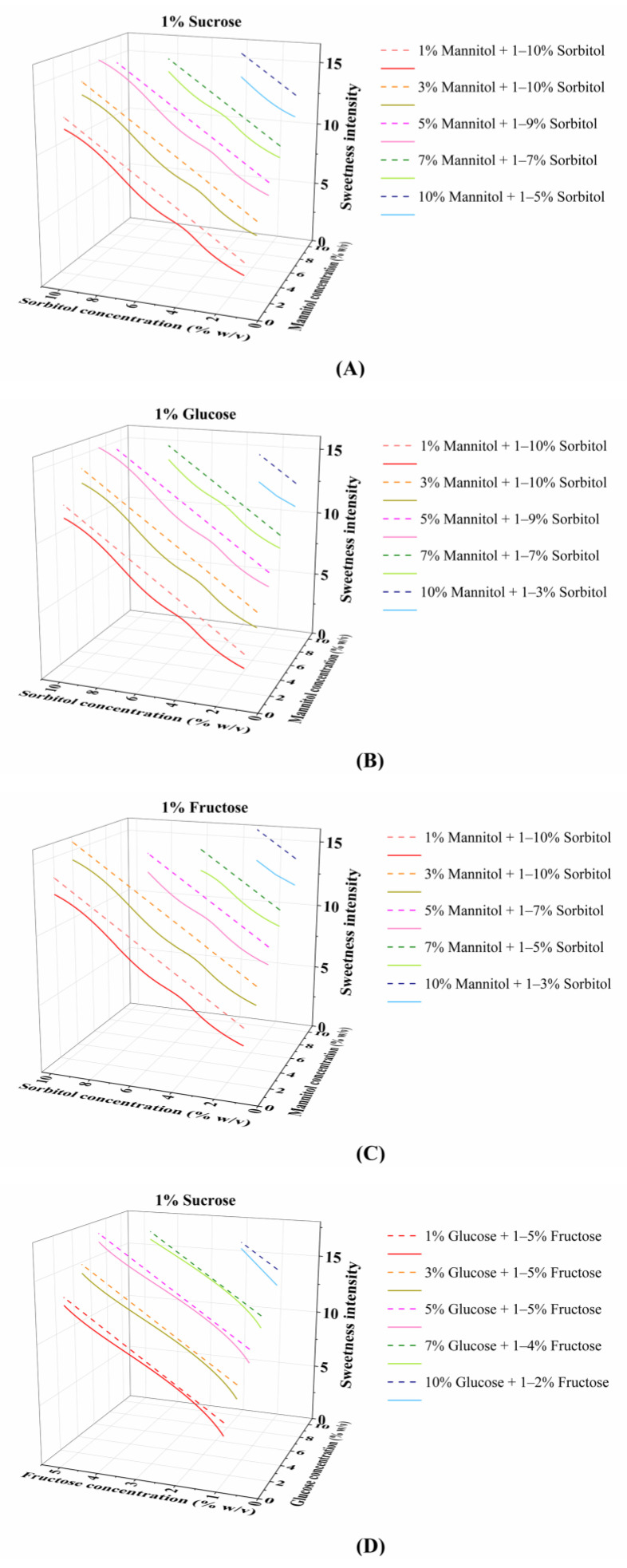
Concentration–sweetness intensity curves of ternary sweeteners within a specific concentration range: (**A**) sucrose, mannitol, and sorbitol; (**B**) glucose, mannitol, and sorbitol; (**C**) fructose, mannitol, and sorbitol; (**D**) sucrose, glucose, and fructose. The curves were generated by fixing the concentration of one sweetener while varying the concentrations of the other two. The dashed lines represent the sweetness intensity of ternary mixtures, and the solid lines represent the direct sum of the sweetness intensities of the individual components. A synergistic effect is indicated when the dashed line of the same color lies above the solid line.

The optimized SVR model was employed to predict the sweetness intensity of ternary sweetener mixtures across a concentration range relevant to typical beverage formulations. To elucidate the quantitative concentration–sweetness interaction relationships in multi-component systems, representative concentration combinations of ternary mixtures were selected for plotting concentration–sweetness intensity relationship curves (Figure 6). These curves were constructed using a fixed-concentration approach: the concentration of one sweetener was held constant while systematically varying the concentrations of the remaining two components.

Under low-concentration conditions, the synergistic effect of ternary sweetener mixtures was particularly pronounced, showing a clear concentration-dependent pattern. When all components were at 1.0%, the mixtures of one sugar and two polyols (Figure 6A–C) exhibited prominent synergism with an increment range of 35.9–42.3%, among which the glucose–mannitol–sorbitol mixture achieved the highest increment. These synergistic increments were all higher than the maximum synergistic increment of binary sweetener mixtures within the same low concentration range (1.0–2.0%), further confirming the synergistic advantage of ternary mixtures over binary mixtures at low concentrations. In contrast, when all three sugars were at 1.0%, the sweetness intensity increased by only 27.7%, indicating weak synergism (Figure 6D). These results aligned with the well-established notion that low concentrations favor stronger synergism in binary mixtures [45] and echoed the sugar–polyol synergistic trend observed in the binary mixtures of this study, further verifying that low concentrations represent the optimal range for ternary synergism. Moreover, the data demonstrate that the synergistic advantage of sugar–polyol mixtures is amplified in ternary mixtures. Building on the findings of Schiffman et al. [17], who reported that ternary mixtures derived from binary synergistic mixtures exhibited higher maximum synergism than binary mixtures, this study further confirms that mixtures of a sugar and two polyols exhibit stronger synergistic effects than other ternary blends, thereby filling the research gap in quantifying the interaction intensity of specific component mixtures. This synergistic advantage may originate from the complementary binding of sugars and polyols at the sweet taste receptor. Based on the mechanistic studies demonstrating the existence of specific binding hotspots for maltitol/lactitol at the receptor [46], we hypothesize that sugar molecules, typified by sucrose, may preferentially occupy the core VFT sites, whereas the isomers mannitol and sorbitol may bind to distinct allosteric sites by virtue of their structural characteristics, thereby forming a non-competitive multi-binding network. This theoretical model suggests that polyols can stabilize the active conformation of the receptor through spatial complementarity, thus providing a plausible molecular mechanism for the enhanced synergistic signal amplification observed in the sugar–polyol ternary mixtures.

When all components in the ternary sweetener mixtures were at concentrations higher than the low-concentration level (1.0%), their synergistic effects were significantly lower than those under low-concentration conditions, and the influence of sweetener types on synergistic differences was more pronounced. The mixtures of one sugar and two polyols exhibited significantly superior synergism compared to the mixtures of three sugars. Specifically, the synergism of mixtures of one sugar and two polyols gradually decreased with increasing concentration, showing an increment range of 8.2–19.6% at 3.0% (Figure S4A,C,G) and decreasing to 9.3–10.6% at 5.0% (Figure S4B,D). In contrast, the mixtures of three sugars showed overall weak synergism that tended to diminish with increasing concentration. For example, their synergistic increment decreased from 14.7% (3.0% sucrose, 3.0% glucose, 1.0% fructose) to 5.3% when fructose increased to 4.0% (Figure S4I), and further dropped to 0.8% in the mixture containing 5.0% sucrose, 7.0% glucose, and 2.0% fructose (Figure S4J). This trend of synergism decreasing with increasing concentration is consistent with that in binary mixtures, further confirming the synergistic advantage of sugar–polyol mixtures over sugar–sugar mixtures. From a molecular mechanism perspective, the binding pockets of sucrose, glucose, and fructose overlap in the VFT domain of the T1R2/T1R3 receptor, which is prone to causing site competition and weakening synergistic effects. In contrast, mixtures of one sugar and two polyols exhibit spatial complementarity in receptor binding, which can reduce competition, optimize receptor conformation, and enhance synergistic effects [14,17]. The conclusion proposed by Liu et al. [46] that polyols preferentially target the T1R2 VFD and form spatial complementarity with sugars directly supports the above mechanism. Additionally, mannitol and sorbitol have lower sweetness intensities than glucose, fructose, and sucrose when used alone. Sucrose is essentially a disaccharide composed of one molecule of glucose and one molecule of fructose, which explains why the mixtures of three sugars exhibit weak synergistic effects: the overlapping binding sites of glucose and fructose lead to competitive inhibition, thus reducing receptor activation efficiency.

Beyond the molecular mechanisms and concentration-dependent patterns discussed above, the synergistic characteristics of the sweetener mixtures identified in this study hold substantial economic significance for the food and beverage industry. Based on the market costs of sweeteners, the binary and ternary sugar–polyol mixtures can effectively optimize the raw material cost structure while achieving the desired sweetness intensity. Leveraging their pronounced synergistic effects, these mixtures can reduce the dosage of high-cost sweeteners, thereby realizing an overall reduction in the total cost of the sweetening system. A comparison of the synergistic patterns between sugar–polyol mixtures (Figure 6A,B) and mixtures of three sugars (Figure 6D) shows that the former exhibits stronger and more stable synergism. This distinctive feature not only ensures sweetness consistency but also has the potential to create unique taste profiles that differ from those of conventional sucrose/high-fructose corn syrup (HFCS)-based formulations, thus expanding the application scenarios of such mixture systems in low-sugar beverages and similar products.

## 4. Conclusions

This study combined sensory experiments with machine learning to establish prediction models for the sweetness intensity of binary and ternary sweetener mixtures. The MLP model for binary mixed sweeteners (R^2^ = 0.9828) and the SVR model for ternary mixed sweeteners (R^2^ = 0.9825) can effectively predict the sweetness intensity, offering a novel technical approach to clarify the quantitative relationship between sweetener concentration and sweetness intensity. Notably, ternary mixtures of one sugar and two polyols exhibit stronger synergism than binary mixtures at low concentrations. These models provide a scientific basis for exploring synergistic/antagonistic laws in multi-component sweetener blends, optimizing blending schemes, and determining the optimal concentration range for beverage formulations. Additionally, the methodological framework established in this study not only provides a reliable tool for the quantitative analysis of natural sweetener blends but can also be extended to the analysis of blending systems involving high-intensity sweeteners (e.g., Ace-K, Reb A) as well as different taste substances (e.g., sweet–acid, sweet–salty blends). Overall, this work offers new technical support for the intelligent sensory quantification of complex taste combinations and the intelligent formulation design of low-sugar beverages.

## Figures and Tables

**Table 1:** The concentrations of each component set in the binary and ternary sweeteners.

Sweetener	Binary Combination Concentrations (% *w*/*v*)					Ternary Combination Concentrations (% *w*/*v*)					
Sucrose	1.0%	3.0%	5.0%	7.0%	10.0%	1.0%	2.0%	3.0%	5.0%	10.0%	/
Glucose	1.0%	3.0%	5.0%	7.0%	10.0%	1.0%	2.0%	3.0%	5.0%	10.0%	/
Fructose	1.0%	2.0%	3.0%	4.0%	5.0%	0.5%	1.0%	1.5%	2.0%	3.0%	5.0%
Mannitol	1.0%	3.0%	5.0%	7.0%	10.0%	1.0%	2.0%	3.0%	5.0%	10.0%	/
Sorbitol	1.0%	3.0%	5.0%	7.0%	10.0%	1.0%	2.0%	3.0%	5.0%	10.0%	/

**Table 2:** Definitions of partial molecular descriptors in the dataset.

Term	Definition
Sucrose concentration	Weight/volume percentage (%) of sucrose in the sweetener mixture
Glucose concentration	Weight/volume percentage (%) of glucose in the sweetener mixture
Fructose concentration	Weight/volume percentage (%) of fructose in the sweetener mixture
Mannitol concentration	Weight/volume percentage (%) of mannitol in the sweetener mixture
Sorbitol concentration	Weight/volume percentage (%) of sorbitol in the sweetener mixture
Mol1	Sucrose
Mol2	Glucose
Mol3	Fructose
Mol4	Mannitol
Mol5	Sorbitol
qed	Quantitative index evaluating the drug-likeness of molecules based on key physicochemical properties
NumRotatableBonds	Number of rotatable bonds, characterizing molecular flexibility
MinEStateIndex	Minimum electrotopological state index, reflecting atomic electron density
Chi4n	Fourth-order molecular connectivity index, describing topological complexity
Chi0v	Zero-order molecular connectivity index, characterizing basic topological features
MinAbsEStateIndex	Minimum absolute electrotopological state index, reflecting local electron distribution
BCUT2D_LOGPHI	2D BCUT logarithmic phi index, distinguishing molecular physicochemical properties
VSA_EState8	E-state-surface area parameter, characterizing regional electron distribution
Chi2n	Second-order molecular connectivity index, describing short-range topological features
LabuteASA	Labute accessible surface area, correlating with molecular hydrophilicity–hydrophobicity
PEOE_VSA1	Charge-surface area parameter, reflecting spatial charge distribution
MaxAbsEStateIndex	Maximum absolute electrotopological state index, characterizing electron distribution heterogeneity
HeavyAtomMolWt	Heavy atom molecular weight, reflecting the mass of molecular core skeleton
SMR_VSA6	Molar refractivity-surface area parameter, characterizing polarizability distribution
MolLogP	Logarithm of molecular octanol-water partition coefficient, characterizing molecular lipophilicity
EState_VSA1	E-state-surface area parameter, reflecting local electron-spatial features
NumAtomStereoCenters	Number of atomic stereocenters, characterizing stereoisomeric complexity
Chi3v	Third-order molecular connectivity index, describing long-range topological branching features

**Table 3:** Performance evaluation results of regression models for binary sweeteners based on all molecular descriptors.

Algorithm	Train R^2^	Test R^2^	Test MSE	Test MAE	Test RMSE
SVR	0.9772	0.9805	0.3021	0.4253	0.5496
XGBoost	0.9947	0.9800	0.3111	0.4098	0.5578
MLP	0.9941	0.9792	0.3225	0.4367	0.5679
GBDT	0.9920	0.9787	0.3307	0.4462	0.5751
RF	0.9172	0.8548	2.2533	1.3080	1.5011
LightGBM	0.8661	0.8242	2.7280	1.3282	1.6517
AdaBoost	0.7054	0.6801	4.9639	1.9793	2.2280

**Table 4:** Performance evaluation results of regression models for ternary sweeteners based on all molecular descriptors.

Algorithm	Train R^2^	Test R^2^	Test MSE	Test MAE	Test RMSE
SVR	0.9817	0.9824	0.1985	0.3416	0.4456
XGBoost	0.9955	0.9805	0.2203	0.3570	0.4694
GBDT	0.9955	0.9785	0.2428	0.3850	0.4928
MLP	0.9937	0.9313	0.7771	0.5684	0.8815
RF	0.9559	0.8935	1.2043	0.8688	1.0974
LightGBM	0.8834	0.7601	2.7117	1.1836	1.6467
AdaBoost	0.6280	0.5922	4.6092	1.8567	2.1469

## Data Availability

The original contributions presented in this study are included in the article/Supplementary Material. Further inquiries can be directed to the corresponding authors.

## Supplementary Materials

The following supporting information can be downloaded at: https://www.mdpi.com/article/10.3390/foods15010167/s1, Figure S1: Sweetness discriminatory ability (A), repeatability (B), and consistency (C) of the sensory panel for different samples. Figure S2: The process of calculating molecular descriptors of five sweeteners using RDKit. Figure S3: Concentration–sweetness intensity curves of binary sweeteners within a specific concentration range: (A) glucose and sorbitol; (B) fructose and sorbitol; (C) sucrose and glucose; (D) sucrose and fructose; (E) glucose and mannitol; (F) fructose and mannitol. Figure S4: Concentration–sweetness intensity curves of ternary sweeteners within a specific concentration range: (A) and (B) sucrose, mannitol, and sorbitol; (C) and (D) glucose, mannitol, and sorbitol; (E), (F), (G) and (H) fructose, mannitol, and sorbitol; (I) and (J) sucrose, glucose, and fructose. Table S1: Concentration settings of five individual sweeteners. Table S2: All molecular descriptors of five sweeteners. Table S3: Hyperparameters used in grid search for different machine learning algorithms. Table S4: SHAP contributions of all molecular descriptors for the four binary sweetener models (MLP, GBDT, XGBoost, and SVR). Table S5: Complete SHAP importance ranking of all molecular descriptors for binary sweeteners. Table S6: SHAP contributions of all molecular descriptors for the four ternary sweetener models (MLP, GBDT, XGBoost, and SVR). Table S7: Complete SHAP importance ranking of all molecular descriptors for ternary sweeteners.

## Abbreviations

Ace-K	Acesulfame-K
NHDC	Neohesperidin dihydrochalcone
Reb A	Rebaudioside A
ML	Machine learning
SHAP	Shapley Additive exPlanations
SMILES	Simplified molecular input line entry system
AdaBoost	Adaptive Boosting
LightGBM	Light Gradient Boosting Machine
RF	Random Forest
GBDT	Gradient Boosting Decision Tree
MLP	Multilayer Perceptron
XGBoost	eXtreme Gradient Boosting
SVR	Support Vector Regression
RMSE	Root mean square error
MSE	Mean square error
MAE	Mean absolute error
Qed	Quantitative estimate of drug-likeness
VFT	Venus flytrap
