# Supplementary material for: Predicting Sweetness Intensity and Uncovering Quantitative Interactions of Mixed Sweeteners: A Machine Learning Approach
Source: Foods. 2026 Jan 4;15(1):167. doi: 10.3390/foods15010167 (PMC12785889; doi:10.3390/foods15010167)
Supplement: Supplementary file 1 [file foods-15-00167-s001.zip › Supplementary File(1).pdf]

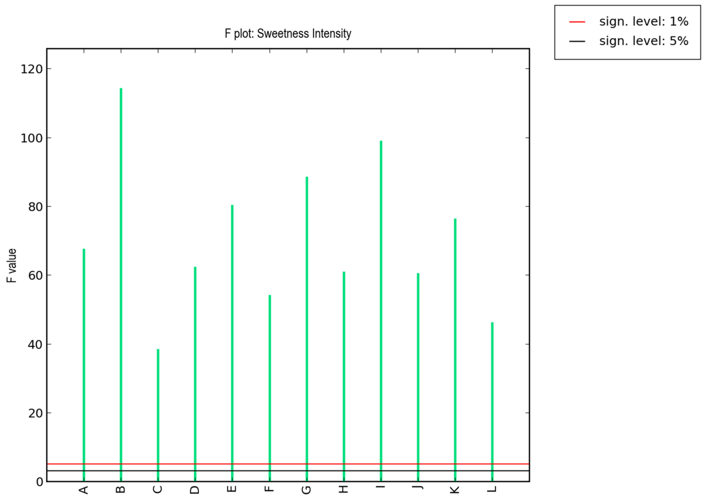

(A)

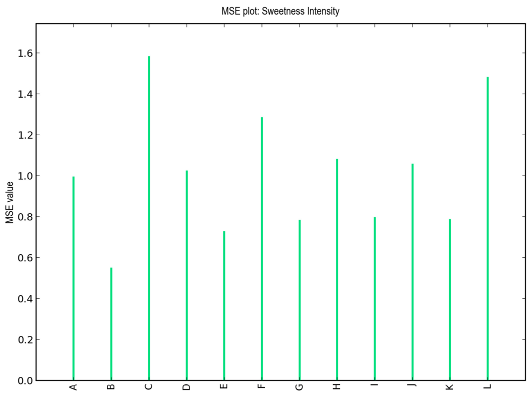

(B)

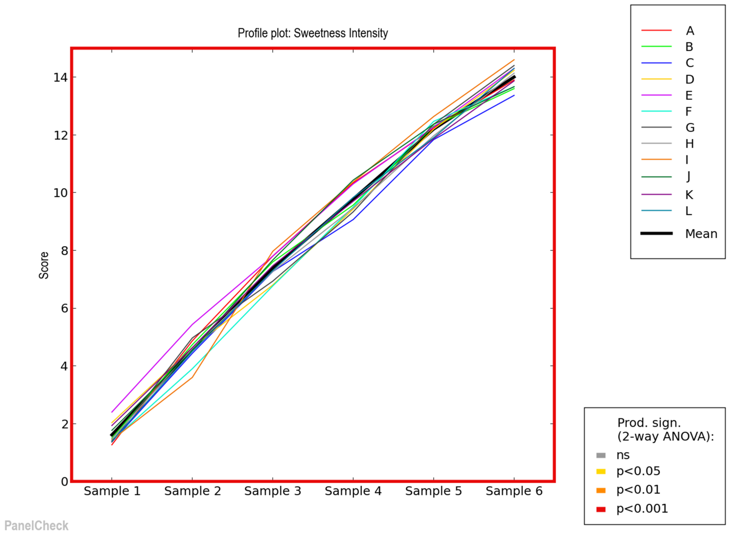

(C)

Figure S1. Sweetness discriminatory ability (A), repeatability (B), and consistency (C) of the sensory panel for different samples.

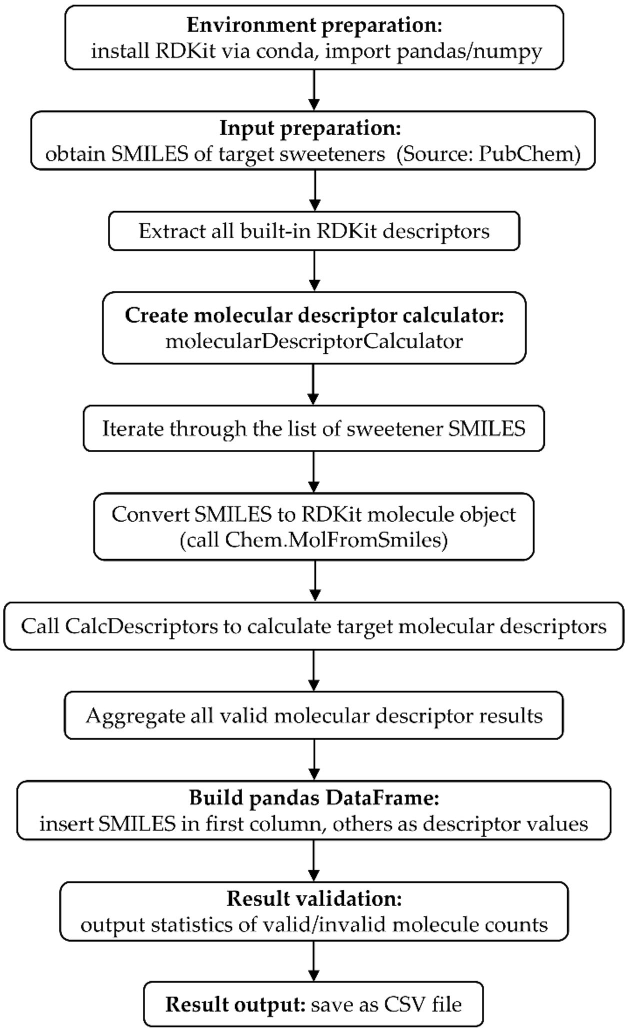

Figure S2. The process of calculating molecular descriptors of five sweeteners using RDKit.

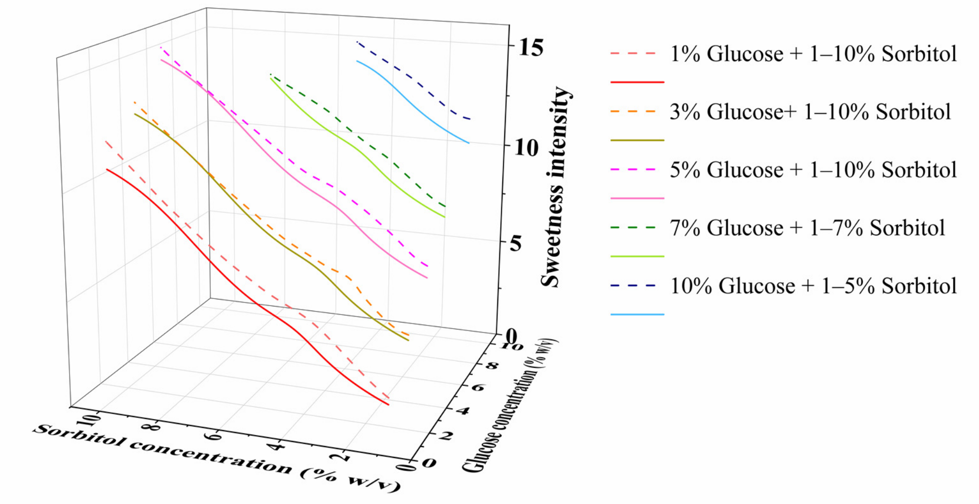
1% Glucose + 1–10% Sorbitol
3% Glucose+ 1–10% Sorbitol
5% Glucose + 1–10% Sorbitol
7% Glucose + 1–7% Sorbitol
10% Glucose + 1–5% Sorbitol
Sweetness intensity
Sorbitol concentration (% w/v)
Glucose concentration (% w/v)

(A)

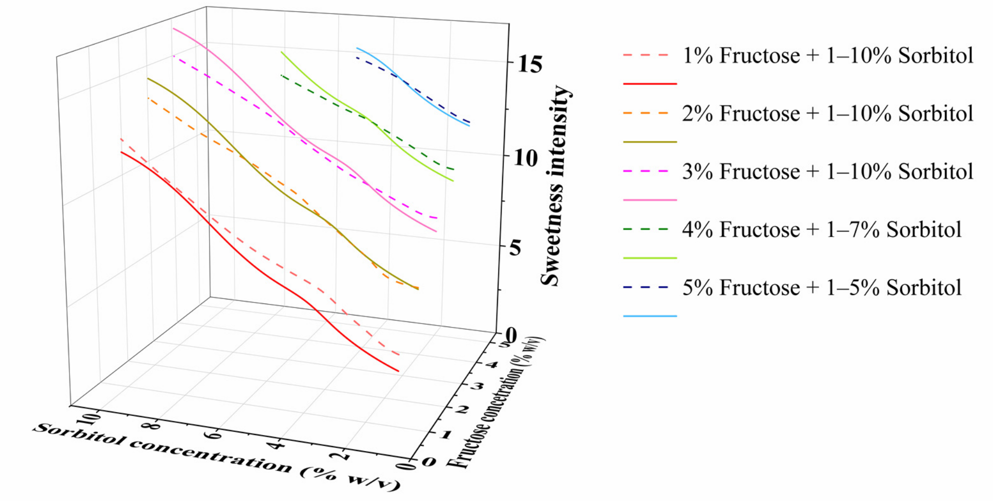
1% Fructose + 1–10% Sorbitol
2% Fructose + 1–10% Sorbitol
3% Fructose + 1–10% Sorbitol
4% Fructose + 1–7% Sorbitol
5% Fructose + 1–5% Sorbitol
Sweetness intensity
Sorbitol concentration (% w/v)
Fructose concentration (% w/v)

(B)

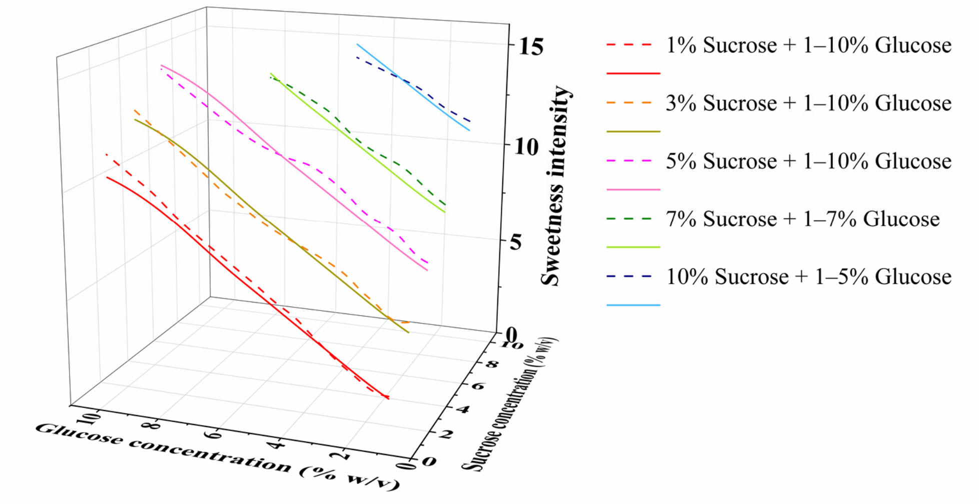
1% Sucrose + 1–10% Glucose
3% Sucrose + 1–10% Glucose
5% Sucrose + 1–10% Glucose
7% Sucrose + 1–7% Glucose
10% Sucrose + 1–5% Glucose
Sweetness intensity
Glucose concentration (% w/v)
Sucrose concentration (% w/v)

(C)

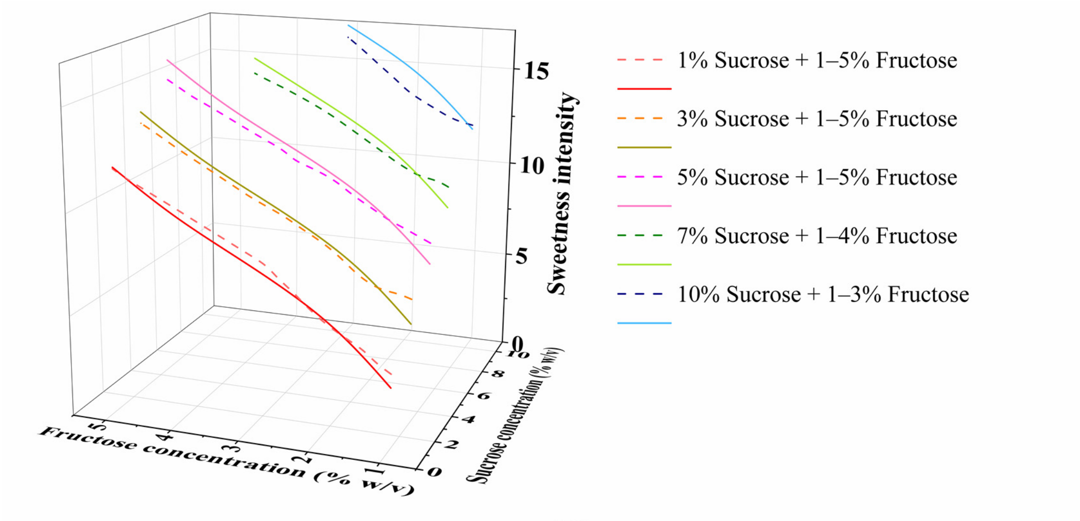

(D)

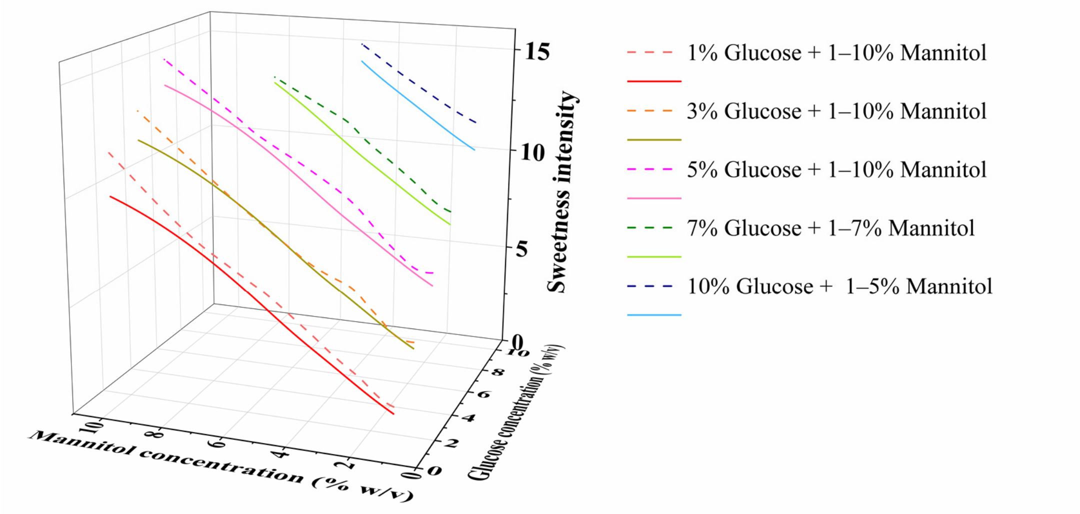

(E)

Figure S3. Concentration-sweetness intensity curves of binary sweeteners within a specific concentration range: (A) glucose and sorbitol; (B) fructose and sorbitol; (C) sucrose and glucose; (D) sucrose and fructose; (E) glucose and mannitol; (F) fructose and mannitol. The dashed lines represent the sweetness intensity of binary mixtures, and the solid lines represent the direct sum of the sweetness intensities of the individual components. A synergistic effect is indicated when the dashed line of the same color lies above the solid line.

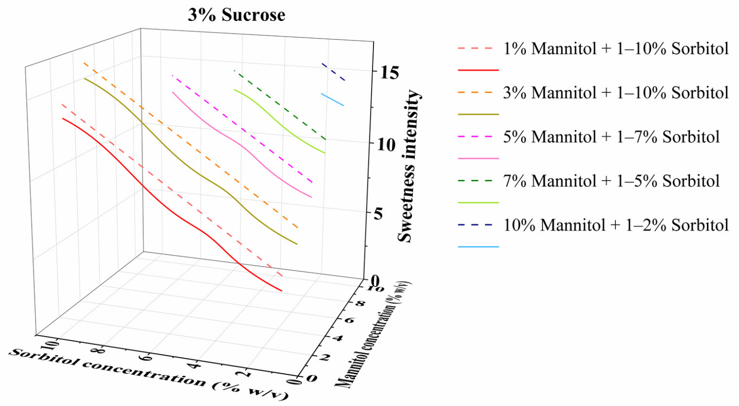
3% Sucrose
1% Mannitol + 1–10% Sorbitol
3% Mannitol + 1–10% Sorbitol
5% Mannitol + 1–7% Sorbitol
7% Mannitol + 1–5% Sorbitol
10% Mannitol + 1–2% Sorbitol
Sweetness intensity
Sorbitol concentration (% w/v)
Mannitol concentration (% w/v)

(A)

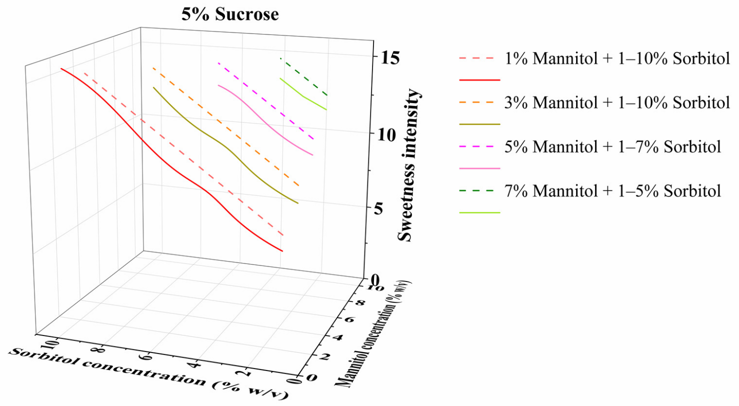
5% Sucrose
1% Mannitol + 1–10% Sorbitol
3% Mannitol + 1–10% Sorbitol
5% Mannitol + 1–7% Sorbitol
7% Mannitol + 1–5% Sorbitol
Sweetness intensity
Sorbitol concentration (% w/v)
Mannitol concentration (% w/v)

(B)

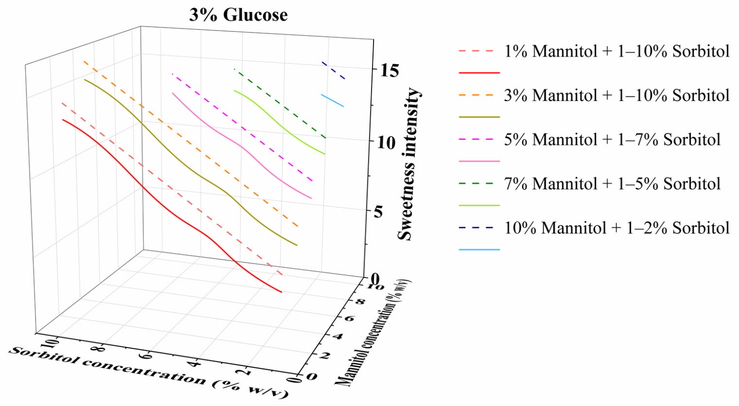
3% Glucose
1% Mannitol + 1–10% Sorbitol
3% Mannitol + 1–10% Sorbitol
5% Mannitol + 1–7% Sorbitol
7% Mannitol + 1–5% Sorbitol
10% Mannitol + 1–2% Sorbitol
Sweetness intensity
Sorbitol concentration (% w/v)
Mannitol concentration (% w/v)

(C)

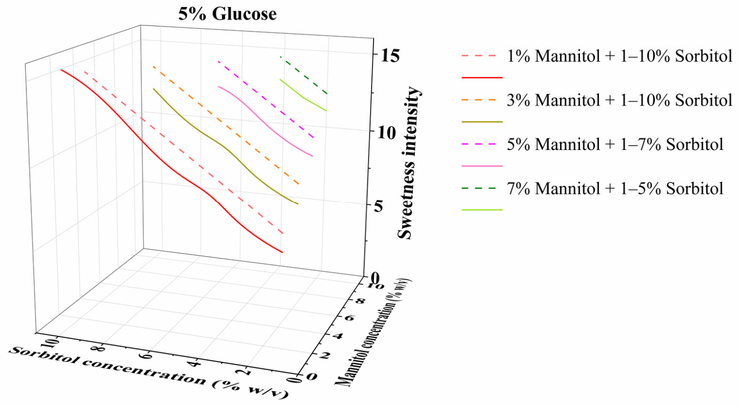
5% Glucose
1% Mannitol + 1–10% Sorbitol
3% Mannitol + 1–10% Sorbitol
5% Mannitol + 1–7% Sorbitol
7% Mannitol + 1–5% Sorbitol
Sweetness intensity
Sorbitol concentration (% w/v)
Mannitol concentration (% w/v)

(D)

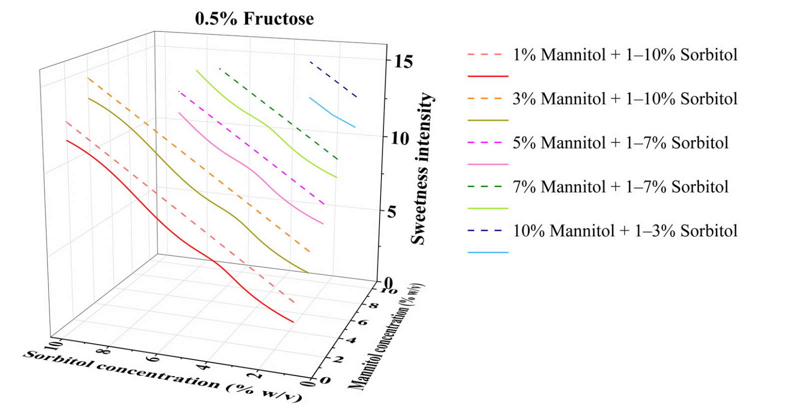
0.5% Fructose
1% Mannitol + 1–10% Sorbitol
3% Mannitol + 1–10% Sorbitol
5% Mannitol + 1–7% Sorbitol
7% Mannitol + 1–7% Sorbitol
10% Mannitol + 1–3% Sorbitol
Sweetness intensity
Sorbitol concentration (% w/v)
Mannitol concentration (% w/v)

(E)

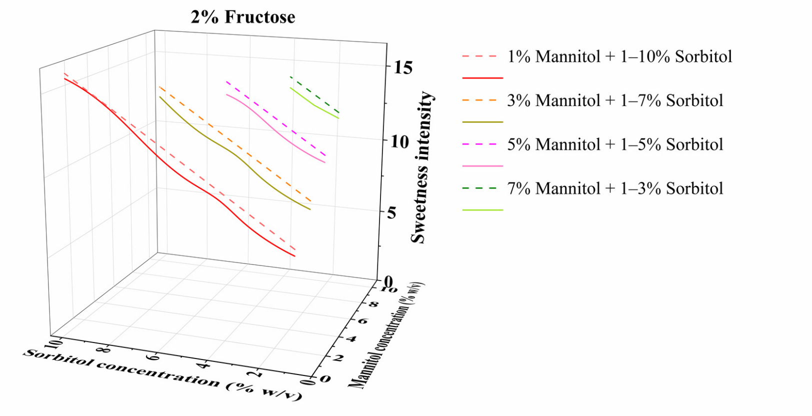
2% Fructose
1% Mannitol + 1–10% Sorbitol
3% Mannitol + 1–7% Sorbitol
5% Mannitol + 1–5% Sorbitol
7% Mannitol + 1–3% Sorbitol
Sweetness intensity
Sorbitol concentration (% w/v)
Mannitol concentration (% w/v)

(F)

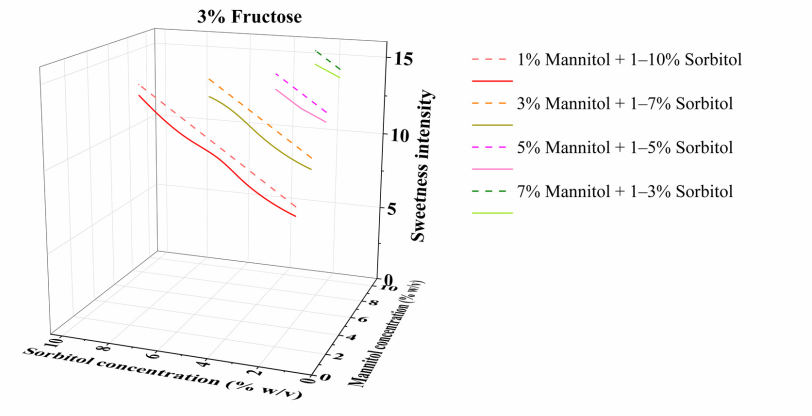
3% Fructose
1% Mannitol + 1–10% Sorbitol
3% Mannitol + 1–7% Sorbitol
5% Mannitol + 1–5% Sorbitol
7% Mannitol + 1–3% Sorbitol
Sweetness intensity
Sorbitol concentration (% w/v)
Mannitol concentration (% w/v)

(G)

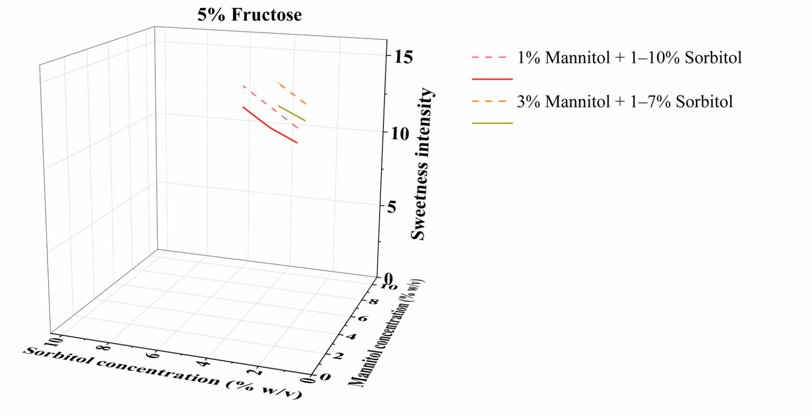
5% Fructose
1% Mannitol + 1–10% Sorbitol
3% Mannitol + 1–7% Sorbitol
Sweetness intensity
Sorbitol concentration (% w/v)
Mannitol concentration (% w/v)

(H)

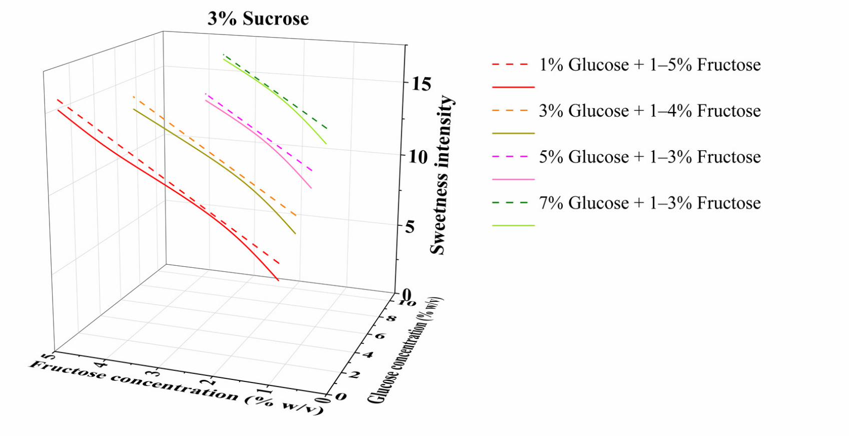

(I)

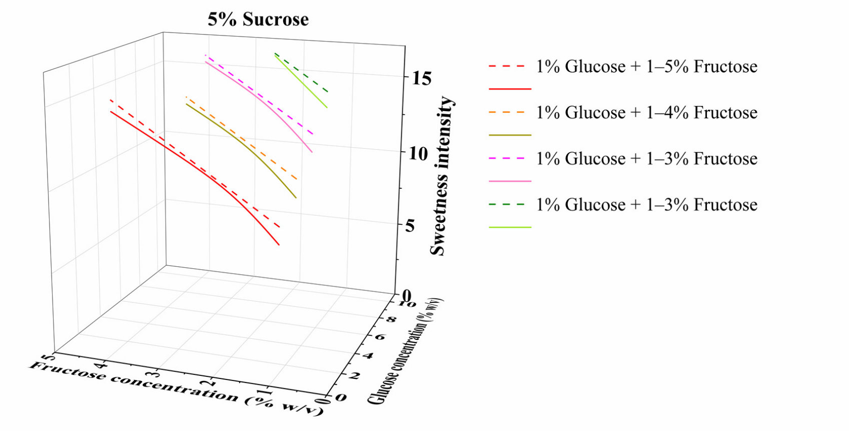

(J)

Figure S4. Concentration-sweetness intensity curves of ternary sweeteners within a specific concentration range: (A) and (B) sucrose, mannitol, and sorbitol; (C) and (D) glucose, mannitol, and sorbitol; (E), (F), (G) and (H) fructose, mannitol, and sorbitol; (I) and (J) sucrose, glucose, and fructose. The curves were generated by fixing the concentration of one sweetener while varying the concentrations of the other two. The dashed lines represent the sweetness intensity of ternary mixtures, and the solid lines represent the direct sum of the sweetness intensities of the individual components. A synergistic effect is indicated when the dashed line of the same color lies above the solid line.

Table S1. Concentration settings of five individual sweeteners.

Sweetener	Concentration (% w/v)									
Sucrose	1.0%	2.0%	3.0%	4.0%	5.0%	6.0%	7.0%	8.0%	9.0%	10.0%
Glucose	1.0%	2.0%	3.0%	4.0%	5.0%	6.0%	7.0%	8.0%	9.0%	10.0%
Fructose	0.5%	1.0%	1.5%	2.0%	2.5%	3.0%	3.5%	4.0%	4.5%	5.0%
Mannitol	1.0%	2.0%	3.0%	4.0%	5.0%	6.0%	7.0%	8.0%	9.0%	10.0%
Sorbitol	1.0%	2.0%	3.0%	4.0%	5.0%	6.0%	7.0%	8.0%	9.0%	10.0%

Table S3. Hyperparameters used in grid search for different machine learning algorithms.

Model	Hyperparameters used in grid search	
	**Regression models for binary sweeteners**	**Regression models for ternary sweeteners**
AdaBoost	estimator_max_depth: 3, estimator_min_samples_split: 2, learning_rate: 0.3, n_estimators: 300	estimator_max_depth: 3, estimator_min_samples_split: 2, learning_rate: 0.3, n_estimators: 300
LightGBM	colsample_bytree: 1, learning_rate: 0.05, max_depth: -1, n_estimators: 200, num_leaves: 31, subsample: 0.8	colsample_bytree: 1, learning_rate: 0.05, max_depth: -1, n_estimators: 200, num_leaves: 31, subsample: 0.8
RF	Bootstrap: True, max_depth: 20, max_features: 'sqrt', min_samples_leaf: 2, min_samples_split: 5, n_estimators: 100, oob_score: True, random_state: 60	Bootstrap: True, max_depth: 20, max_features: 'sqrt', min_samples_leaf: 2, min_samples_split: 5, n_estimators: 200, oob_score: True, random_state: 60
GBDT	learning_rate: 0.1, max_depth: 3, min_samples_leaf: 4, min_samples_split: 10, n_estimators: 300, subsample： 0.9	learning_rate: 0.1, max_depth: 3, min_samples_leaf: 4, min_samples_split: 10, n_estimators: 500, subsample： 0.9
MLP	Activation： 'relu', alpha: 0.001, hidden_layer_sizes: (100, 50) (binary), learning_rate_init: 0.001	Activation： 'relu', alpha: 0.0001, hidden_layer_sizes: (50, 50, 50) (ternary), learning_rate_init: 0.001
XGBoost	colsample_bytree: 1.0, learning_rate: 0.2, max_depth: 3, n_estimators: 200, reg_alpha: 0.1, reg_lambda: 1, subsample: 0.8	colsample_bytree: 1.0, learning_rate: 0.2, max_depth: 3, n_estimators: 200, reg_alpha: 0.1, reg_lambda: 0.1, subsample: 0.8
SVR	C: 1, epsilon: 0.01, gamma: 0.0001, kernel: 'linear'	C: 0.1, epsilon: 0.05, gamma: 0.001, kernel: 'linear'
